# Langerhans cell histiocytosis of the rib presenting with pathological fracture: a case report

**DOI:** 10.1186/s13019-020-01368-9

**Published:** 2020-11-23

**Authors:** Tao Zuo, Ping Jiang, Junjie Yu, Ke Zhao, Yong Liu, Baojun Chen

**Affiliations:** 1 Department of Thoracic Surgery, The Central Hospital of Wuhan, Tongji Medical College, Huazhong University of Science and Technology, Wuhan, China; 2 Department of ophthalmology, Zhongnan Hospital of Wuhan University, Wuhan, China

**Keywords:** Langerhans cell Histiocytosis, Rib, Pathological fracture

## Abstract

**Introduction:**

Langerhans cell histiocytosis (LCH) is a rare neoplastic hyperplasia with an unknown etiology. It is clinically rare for patients with solitary rib lesion and pathological fracture; moreover, its diagnosis and treatment are quite difficult. The purpose of this study is to present a case for the pathogenesis, clinical features, imaging, and treatment of this disease.

**Case presentation:**

A 52-year-old female patient complained of left chest pain for one week. CT showed a fracture in the left 5th rib. The rib tumor was then resected and the surrounding muscles and soft tissues were accordingly resected. The patient was diagnosed with pathological rib fracture, and the patient was pathologically diagnosed with LCH. After surgery, no local recurrence or distant metastasis was reported during the two-year follow-up.

**Conclusions:**

LCH should be treated by observation, chemotherapy, radiotherapy, or surgery, or using a combination of several methods. Moreover, primary tumor should be considered when rib fracture without trauma and tumor metastasis.

## Introduction

Langerhans cell histiocytosis (LCH), known as eosinophilic granuloma of bone, is a rare neoplastic hyperplasia having an unknown etiology. LCH is characterized by intense and abnormal proliferation of bone marrow-derived histiocytes (Langerhans cells), leading to its high rate of misdiagnosis and missed diagnosis. LCH has a highly variable clinical presentation, ranging from a single lesion to potentially fatal disseminated disease. It is clinically rare for patients with solitary rib lesion and pathological fracture, and its diagnosis and treatment are quite difficult. In this study, we report a patient with LCH presenting with pathological rib fracture.

## Case presentation

**Fig. 1:**
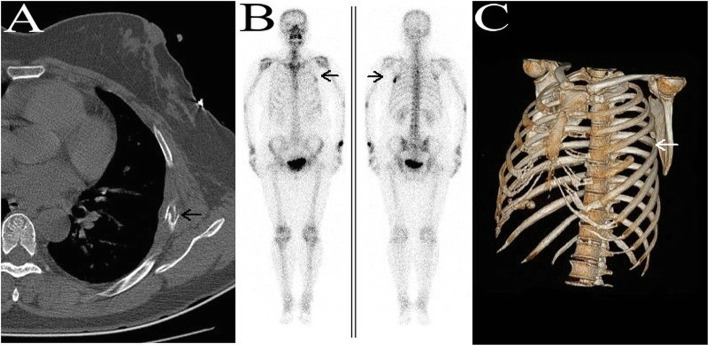
**a**. Preoperative chest CT suggests pathological fracture. **b**. Bone scanning suggests increased local metabolism of the lesion. **c**. Postoperative CT of chest wall reconstruction

**Fig. 2:**
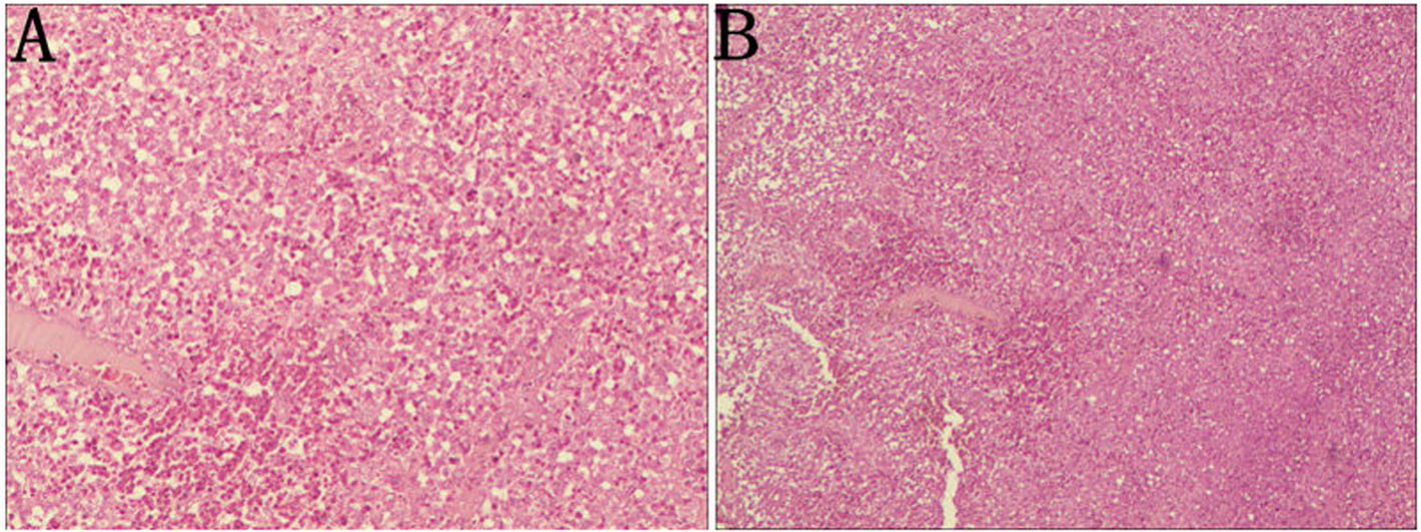
Pathology shows Langerhans cells and mediated inflammatory cells, including lymphocytes, eosinophils and macrophages

**Fig. 3:**
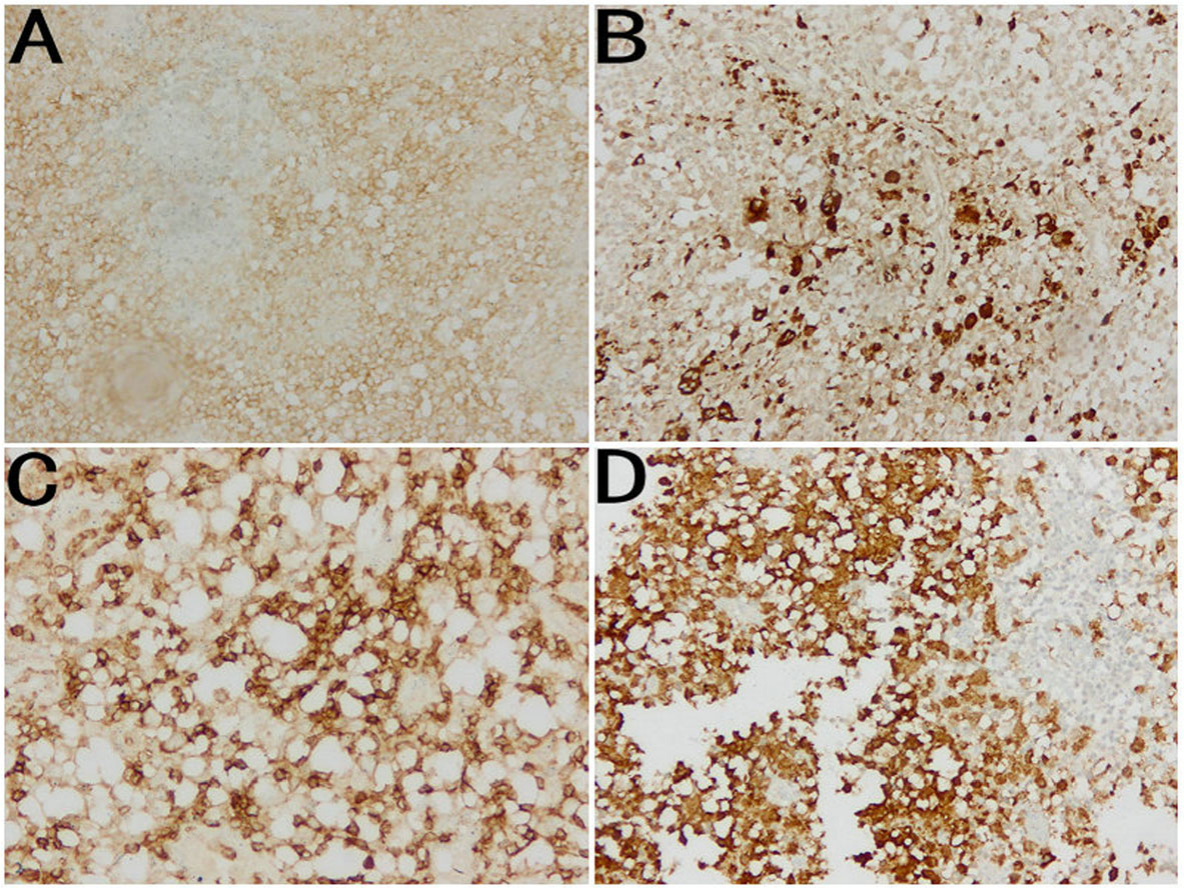
Immunohistochemistry demonstrated CD1a (**a**),CD68 (**b**), LCA (**c**) and S-100 (**d**) are positive

A 52-year-old female patient complained of left chest pain for one week. The patient had no history of trauma or other diseases. Physical examination indicated slight tenderness in the left chest and normal breathing sounds in both lungs. Chest computed tomography (CT) conducted at the outpatient department indicated local bone density reduction along with fracture of the left 5th rib and thickening of the soft tissue adjacent to the chest wall; the possibility of a pathological fracture could not be excluded (Fig. 1a). Thus, relevant examinations were performed. Bone scanning presented abnormal radioactive concentrations in the left 5th rib (Fig. 1b). No abnormalities were then revealed on the head magnetic resonance imaging (MRI) or color doppler ultrasound of the liver, gallbladder, pancreas, and spleen. The patient was diagnosed with a single rib lesion. Because of the reduced effect that surgical resection had on respiratory function, the rib tumor was resected and surrounding muscles and soft tissues were accordingly resected. Postoperative pathology indicated massive Langerhans cell infiltration (Fig. 2). Immunohistochemistry demonstrated CD1a (+) and S-100 (+) (Fig. 3). Therefore, the patient was diagnosed with LCH; after surgery, no local recurrence or distant metastasis was reported during the two-year follow-up (Fig. 1c).

## Discussion

LCH is a clonal proliferative disease caused by the proliferation and aggregation of Langerhans cells (CD207^+^) with abnormal function in single or whole-body tissues and organs [1]. I the general population, its incidence is estimated to be 1 or 2 cases per 1 million individuals, whereas incidence in children aged 1–3 years is even higher at 3–5 cases per 1 million individuals [2, 3]. LCH can involve a single organ, a single system and multiple organs, or multiple systems and multiple organs [4]. Its symptoms range from single self-absorbed organ hyperplasia (the mildest) to systemic infiltrative hyperplasia (the severest). The organs that are always involved include the bone, lung, central nervous system, liver, thymus, skin, and lymph nodes. As for bones, the skull, long bone, and flat bone are the most susceptible. The mortality rate of patients with multiorgan LCH is 10–20% [5]. The clinical manifestations of LCH are nonspecific, and this lack of specificity makes its diagnosis difficult. However, once LCH is considered as a possibility, the diagnosis can then be confirmed by biopsy. Pathology will show pathological Langerhans cells and mediated inflammatory cells, including lymphocytes, eosinophils, and macrophages. S-100 protein, CD1a, and CD207 in Langerhans cells are positive, whereas Birbeck granules under an electron microscope are specific [6, 7].

LCH can be treated by observation, chemotherapy, radiotherapy, or surgery, or using a combination of several methods. Patients with good prognosis may only require follow-up observation or little intervention, whereas patients with poor prognosis should be actively treated. The long-term efficacy and prognosis still require further follow-up observation [8]. A study has demonstrated that only 2 of 61 patients with LCH of single bone had a recurrence after surgical resection [9]. Another study has demonstrated that the four-year survival rate of patients with LCH of single bone is > 90% [10]. Teratogenic surgery is not recommended for patients with a single small lesion because it is always self-limiting. In such cases, the local lesion of the rib is definitively diagnosed via surgical resection, whereas the resection has little effect on the patient’s function. Similar to other benign tumors, surgical removal is effective in this case [11, 12]. Local radiotherapy of bone lesions is only suitable for patients with lesion progression, which may affect the function of important organs. Patients with systemic invasion should be actively treated using chemotherapy, whereas high-risk patients with multisystem diseases accompanied by organ dysfunction should be treated using systemic therapy along with chemotherapy.

Generally, among young adults, the skeletal loads causing rib fractures are attributed to high-energy traumatic events. In older adults, rib fractures often result from falls [13, 14]. Pathological rib fracture is a common manifestation of malignant tumor rib metastasis. In this study, primary tumor of ribs may lead to rib fracture.

## Conclusions

In conclusion, we report a patient with LCH who presented with pathological rib fracture; for this case, surgical removal was effective. However, LCH should be treated by observation, chemotherapy, radiotherapy, or surgery, or using a combination of several methods. Furthermore, primary tumor should be considered when rib fracture without trauma and tumor metastasis.

## Data Availability

Not applicable.
